# The Plant Derived 3-3′-Diindolylmethane (DIM) Behaves as CB_2_ Receptor Agonist in Prostate Cancer Cellular Models

**DOI:** 10.3390/ijms24043620

**Published:** 2023-02-11

**Authors:** Paolo Tucci, Iain Brown, Guy S. Bewick, Roger G. Pertwee, Pietro Marini

**Affiliations:** 1 Department of Clinical and Experimental Medicine, University of Foggia, 71122 Foggia, Italy; 2 Division of Applied Medicine, School of Medicine and Dentistry, Foresterhill, University of Aberdeen, Aberdeen AB25 2ZD, UK; 3 The Institute of Medical Sciences, Foresterhill, University of Aberdeen, Aberdeen AB25 2ZD, UK; 4 Institute of Education in Healthcare and Medical Sciences, Foresterhill, University of Aberdeen, Aberdeen AB25 2ZD, UK

**Keywords:** DIM, CB_2_ cannabinoid receptors, prostate cancer

## Abstract

3-3′-Diindolylmethane (DIM) is a biologically active dimer derived from the endogenous conversion of indole-3-carbinol (I3C), a naturally occurring glucosinolate found in many cruciferous vegetables (i.e., *Brassicaceae*). DIM was the first pure androgen receptor antagonist isolated from the *Brassicaceae* family and has been recently investigated for its potential pharmacological use in prostate cancer prevention and treatment. Interestingly, there is evidence that DIM can also interact with cannabinoid receptors. In this context, by considering the well-known involvement of the endocannabinoid system in prostate cancer, we have pharmacologically characterized the properties of DIM on both CB_1_ and CB_2_ cannabinoid receptors in two human prostate cancer cell lines: PC3 (androgen-independent/androgen receptor negative) and LNCaP (androgen-dependent). In the PC3 cell line, DIM was able to activate CB_2_ receptors and potentially associated apoptotic pathways. On the other hand, although DIM was also able to activate CB_2_ receptors in the LNCaP cell line, no apoptotic effects were observed. Our evidence confirms that DIM is a CB_2_ receptor ligand and, moreover, it has a potential anti-proliferative effect on androgen-independent/androgen receptor-negative prostate cancer cells.

## 1. Introduction

3-*3′*-Diindolylmethane (DIM) is a biologically active dimer derived from the conversion of indole-3-carbinol (I3C). I3C is produced endogenously from naturally occurring glucosinolates (GLs) contained in a wide variety of plant food substances, including members of the family *Cruciferae*, and particularly members of genus *Brassica* [1,2,3]. The amount of GLs in *Brassicaceae* vegetables is 206–3895 mg/kg [4]. Whether the plant tissues are crushed or cooked, an endogenous thioglucosidase (myrosinase) is activated and converts glucosinolates to indoles, principally to I3C [5]. When I3C is orally ingested, due to its chemical instability in acidic conditions, such as in the stomach environment, the compound is promptly condensed into DIM, which is the bioactive product [6], as demonstrated by pharmacokinetics studies conducted in animals [7,8] and human models [9,10]. DIM has been shown to possess hepatoprotective, antioxidant and anticancer properties [11,12,13]. In particular, the anticancer effect of DIM has been positively associated with a reduction of cancer incidence when a regular consumption of cruciferous vegetables is part of the dietary regime [14]. Specifically, DIM can induce apoptosis and can reduce proliferation and metastasis of tumor cells, as well as the inflammation process. Moreover, DIM appears to be a promising agent for the prevention of the recurrence of hormone-dependent cancers, such as prostate cancer [6]. In this context, hormone-dependent prostate cancer represents the sixth leading cause of cancer deaths in males, as well as the second most common form [15]. Indeed, most prostate cancer cases are dependent on androgen at initial stages, and the inhibition of the function of androgen receptors (AR) (i.e., by using anti-androgen drugs), is currently a coadjutant therapeutical approach, along with surgery or radiation, in the treatment of this type of cancer [16]. Moreover, DIM is the first androgen receptor antagonist derived from plants with a binding affinity similar to antiandrogens, such as cyproterone acetate and bicalutamide. In addition, it can downregulate AR signaling to prevent the translocation of these receptors from the cytoplasm to the nucleus [17]. Interestingly, DIM has also demonstrated an ability to antagonize the aryl hydrocarbon receptor [18], as well as modulate cytochrome P450 1A1 activity [19]. Further evidence has also confirmed the protective role of DIM in androgen-independent prostate cancer. For example, microarray analysis conducted on PC3 cells (androgen-independent/androgen receptor negative) showed that many genes involved in the control of carcinogenesis and cell survival are modulated in their expression by indoles, including DIM [20]. Similarly, clinical trial results have demonstrated the therapeutic efficacy of DIM in the treatment of patients with high-grade prostatic intraepithelial neoplasia [21]. DIM has been demonstrated to interact with the endocannabinoid system, by stimulating both CB_1_ and CB_2_ cannabinoid receptors. Specifically, in an experimental model using hCB_1_/CB_2_-CHO transfected cells, DIM was able to interact as a slight inverse agonist on CB_1_ receptors and as a partial agonist on CB_2_ receptors [22]. Therefore, the endocannabinoid system has been proposed as a possible target for the treatment of prostate cancer, and extensive studies have demonstrated the involvement of the system in the pathology. In fact, results from preclinical studies have highlighted altered levels of endocannabinoids in patients’ plasma and identified a correlation between high CB_1_/CB_2_ receptor expression and a poor prognosis [23,24]. In human prostate cancer cell lines (e.g., LNCaP and PC3), higher levels of CB_1_ and CB_2_ receptor expressions are detected in comparison with non-cancerous prostate cells [25], plus elevated levels for the enzymes responsible for endocannabinoid degradation, suggesting a potential role of the endocannabinoid system in prostate cell proliferation [26]. In fact, a number of synthetic and endogenous agonist ligands for CB_1_ and CB_2_ receptors, along with inhibitors of endocannabinoid enzyme degradation, have anticancer effects [27]. In PC3 cells and in primary cultures from benign prostate hyperplasia or prostate cancer, the endocannabinoid system is responsible for decreasing cell viability, mostly through the activation of CB_1_ receptors [28]. Considering the role played by the endocannabinoid system in prostate cancer, and the potential anticancer effect of DIM, the aim of this study was to characterize the ability of DIM to interact with both CB_1_ and CB_2_ receptors and to evaluate any effect on apoptotic processes in cellular models of prostate cancer, such as PC3 (androgen-independent human prostate cancer cells) and LNCaP (androgen-dependent human prostate cancer cell line) that naturally express the endocannabinoid system [29,30].

## 2. Results

### 2.1. DIM Is a CB_2_ Receptor Agonist in Both PC3 and LNCaP Cells

**Figure 1:**
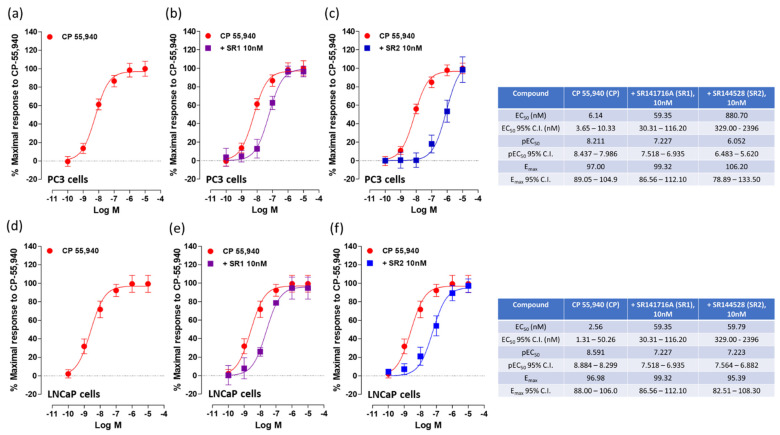
Effects of DIM and CP 55,940 on [^35^S]GTPγS in PC3 cell membranes. The effects of CP 55,940 and DIM (**a**,**d** respectively) were also tested in the presence of CB_1_ receptor antagonist SR1 (**b**,**e**) and CB_2_ receptor antagonist SR2 (**c**,**f**). Each data point is the mean percentage value ± S.E.M. (*n* = 15). EC_50_, E_max_ and pEC_50_ values and 95% confidence intervals are shown in the table.

When tested in PC3 cell membranes, DIM was able to stimulate [^35^S]GTPγS binding (EC_50_: 9.28 nM) showing full agonist behavior (Figure 1a) with a potency similar to that of the full CB_1_/CB_2_ receptor agonist CP 55,940 (EC_50_: 6.14 nM) (Figure 1d). To determine whether the elicited effect of DIM was due to the activation of CB_1_ or CB_2_ receptors, we tested whether the effect of DIM could be antagonized with well-known CB_1_ and CB_2_ receptor antagonists/inverse agonists SR141716A (SR1) and SR144528 (SR2) respectively. SR1 (10 nM) produced no significant dextral shift in the log concentration–response curve (Figure 1b). Conversely, SR2 (10 nM) did produce a significant dextral shift in the log concentration–response curve of DIM (Figure 1c). As a positive control, the effect of CP 55,940 was assayed in the same system producing a marked dextral shift in the log concentration–response dose observed in the presence of SR2 (10 nM) in addition to a slight, but significant, dextral shift in the log concentration–response in the presence of SR1 (10 nM) (Figure 1e,f, respectively).

**Figure 2:**
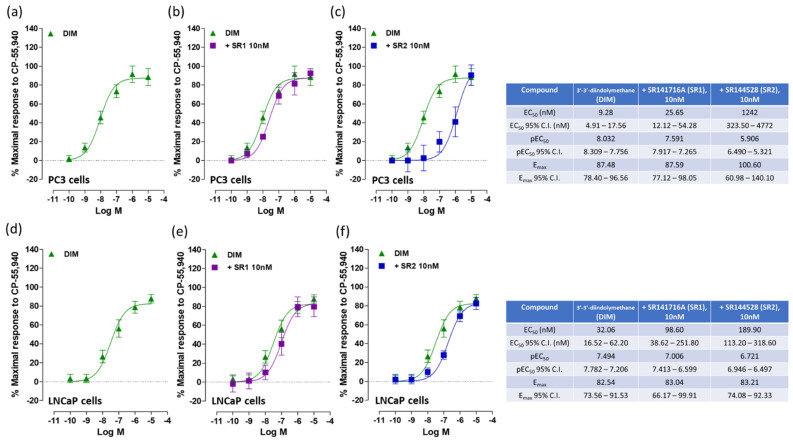
Effects of DIM and CP 55,940 on [^35^S]GTPγS in LNCaP cell membranes. The effects of DIM and CP 55,940 (**a**,**d** respectively) were also tested in the presence of CB_1_ receptor antagonist SR1 (**b**,**e**) and CB_2_ receptor antagonist SR2 (**c**,**f**). Each data point is the mean percentage value ± S.E.M. (*n* = 15). EC_50_, E_max_, pEC_50_ and 95% confidence intervals are shown in the table.

In LNCaP cell membranes, DIM was able to stimulate [^35^S]GTPγS binding (EC_50_: 32.06 nM) with less potency compared to CP 55,940 (EC_50_: 2.56 nM) (Figure 2a,d). As shown in Figure 2c, in the presence of the CB_2_ receptor antagonist SR2 (10 nM), a significant dextral shift in the log concentration–response curve of DIM was observed, whilst no significant effect was observed in presence of the CB_1_ receptor antagonist SR1 (Figure 2b). Since there was no evidence of a significant interaction of DIM with CB_1_ receptors, further experiments with intact cells focused on its actions through CB_2_ receptors.

### 2.2. DIM Induces an Antiproliferative Effect through CB_2_ Receptor Stimulation Only in PC3 Cells

**Figure 3:**
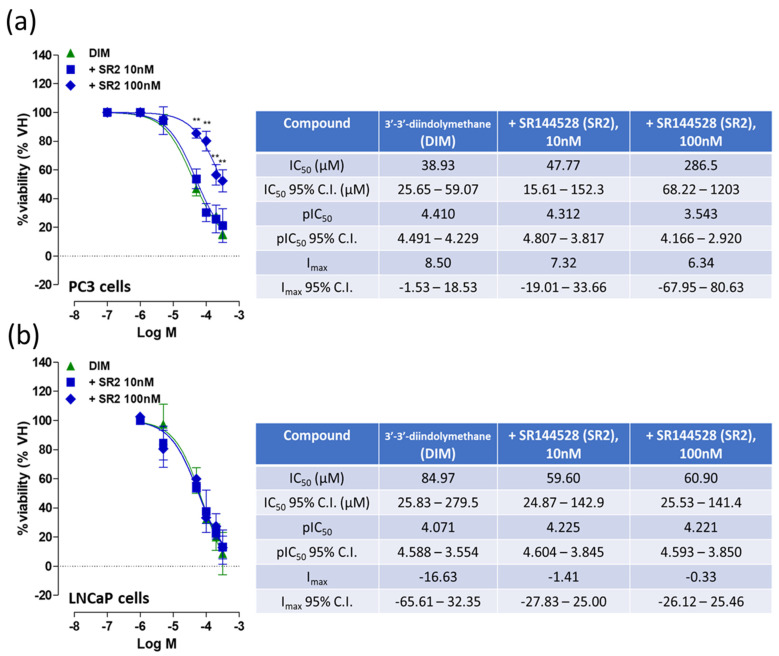
Effects of DIM and different concentrations of the CB_2_ receptor antagonist SR2 on cell viability in PC3 cells (**a**) and LNCaP cells (**b**). Each data point is the mean percentage value ± S.E.M (*n* = 7). IC_50_, pIC_50_ and I_max_ values and 95% confidence intervals are shown in the table. ** *p* < 0.01 (paired Student’s *t*-test).

When tested in the cell viability assay, DIM was able to induce an antiproliferative effect in PC3 cells (IC_50_: 38.93 μM) (Figure 3a). This apoptotic effect was antagonized by the CB_2_ receptor antagonist SR2, with a significant dextral shift in the log concentration–response observed. Interestingly, while DIM also induced an antiproliferative effect in LNCaP cells, no antagonist effect with SR2 was observed (Figure 3b), indicating this was not via CB_2_ receptors, so these were not considered further. To better understand the CB_2_-mediated effects, further experiments were conducted on PC3 cells.

**Figure 4:**
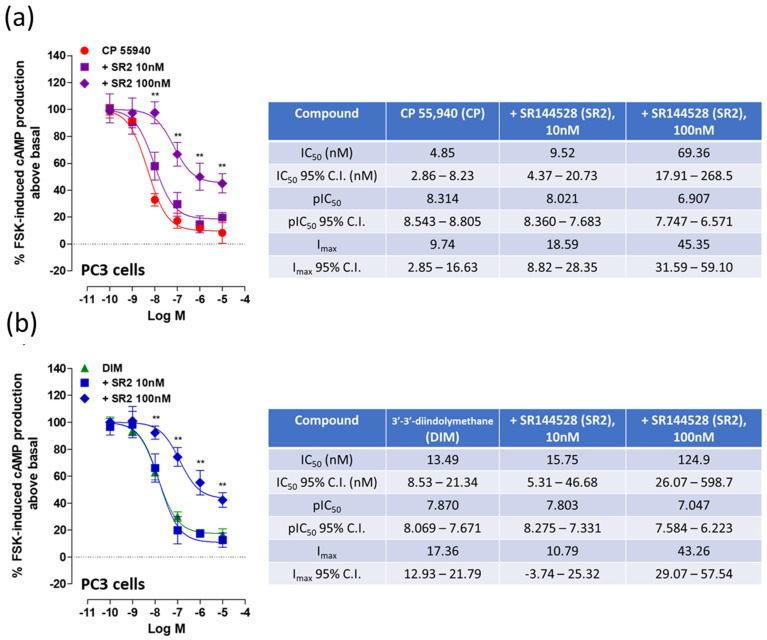
Effects of CP 55,940 (**a**) and DIM (**b**) on FSK-induced cAMP production in PC3 cells. The effects of different concentrations of the CB_2_ receptor antagonist SR2 in presence of CP 55,940 (**a**) or DIM (**b**) are also shown. Each data point is the mean percentage value ± S.E.M. (*n* = 10). IC_50_, pIC_50_ and I_max_ values and 95% confidence intervals are shown in the tables. ** *p* < 0.01 (paired Student’s *t*-test).

In PC3 cells, DIM was able to inhibit FSK-induced cAMP production with a potency in the nanomolar range (IC_50_: 13.49 nM) (Figure 4b), showing a similar effect to the well-known CB_1_/CB_2_ receptor agonist CP 55,940 (IC_50_: 4.85 nM) (Figure 4a). The inhibitory effect of both DIM and CP 55, 940 on FSK-induced cAMP production, was tested in presence of the CB_2_ receptor antagonist SR2. Again, as for the viability assay, when tested at the dose of 10 nM, SR2 did not induce any significant effect on FSK-induced cAMP production inhibition elicited in the presence of both CP 55,940 and DIM. However, previous studies suggest a dextral shift requires a much higher dose of antagonists in an intact cell assay compared to isolated cell membranes [31,32,33]. At the dose of 100 nM, SR2 was indeed able to significantly inhibit both CP 55,940 and DIM effects on FSK-induced cAMP production (Figure 4a,b). In contrast, the CB_1_ receptor antagonist SR1 had no effect on DIM’s inhibition of FSK-induced cAMP production.

### 2.3. DIM Induces Changes in pAKT (Reduction) and Cleaved-CASP-3 (Increase) Levels through CB_2_ Receptor Stimulation in PC3 Cell Line

**Figure 5:**
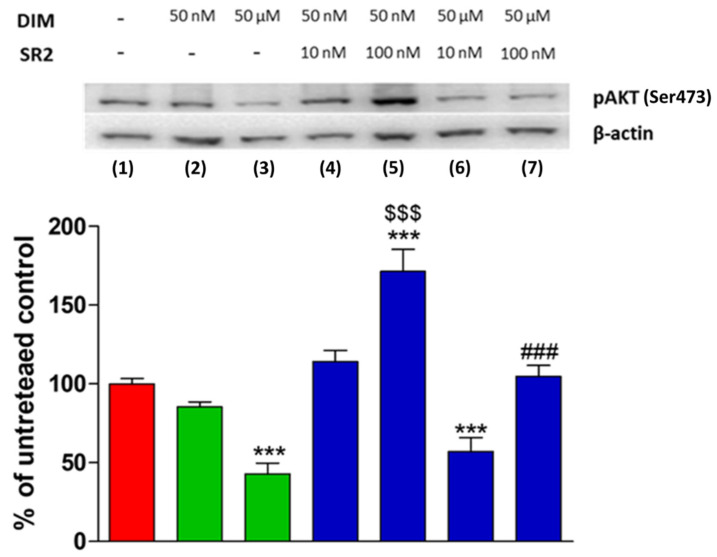
Western blot analysis of phosphor-AKT (Ser473) levels in PC3 cells. The effects of different concentrations of DIM alone and in the presence of different concentrations of the CB_2_ receptor antagonist SR2 are shown. Levels are normalized to actin internal loading control and expressed as a percentage compared to untreated control cells. Each data point is the mean percentage value ± S.E.M. (*n* = 3). *** *p* < 0.001 vs. CTRL (1), $$$ *p* < 0.001 vs. DIM 50 nM (2), ### *p* < 0.001 vs. DIM 50 µM (3). (one-way ANOVA analysis of variance with Tukey’s post hoc analysis).

Since DIM had an antiproliferative effect on PC3 cells, we tested the compound for its ability to modulate the expression of the phosphorylated form of AKT (pAKT) at doses of 50 nM and 50 µM. There was no significant reduction at 50 nM but at the higher dose of 50 µM, a strong and significant reduction of pAKT level was observed (Figure 5, lanes 2 and 3). To further determine if the effect of DIM on pAKT was CB_2_ receptor-mediated, two different doses (10 nM and 100 nM) of the CB_2_ receptor antagonist SR2 were tested. At 10 nM, SR2 also had no significant effect when applied with the ineffective dose of DIM (50 nM; Figure 5, lane 4). However, 100 nM SR2 actually caused a strong increase of pAKT level in the presence of 50 nM DIM (Figure 5, lane 5), indicating a strong inverse agonism effect.

The robust reduction of the pAKT level produced by 50 µM DIM (Figure 5, lane 3) was only marginally, but significantly, reduced by low dose SR2 (Figure 5, lane 6 vs. lane 3), whereas at the higher dose of 100 nM, the CB_2_ receptor antagonist essentially reversed the DIM-induced pAKT level decrease (Figure 5, lane 7).

**Figure 6:**
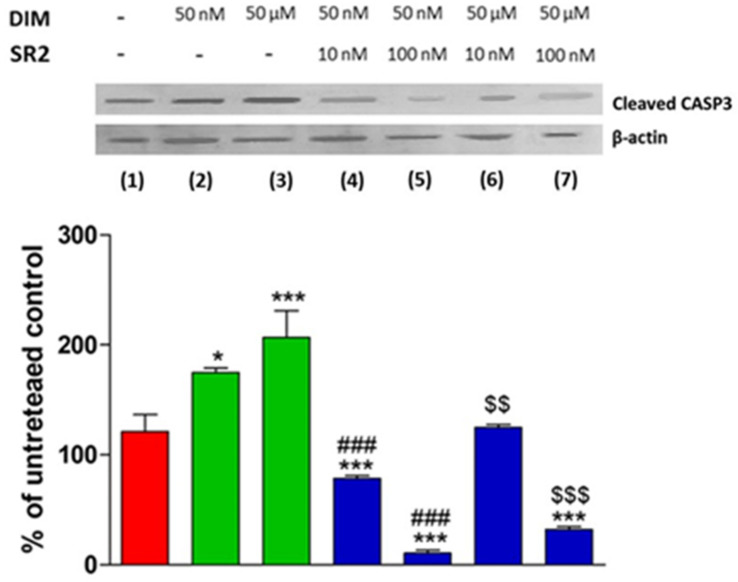
Western blot analysis of cleaved caspase 3 (CASP-3) levels in PC3 cells. The effects of different concentrations of DIM alone and in the presence of different concentrations of the CB_2_ receptor antagonist SR2 are shown. Levels are normalized to actin internal loading control and expressed as a percentage compared to untreated control cells. Each data point is the mean percentage value ± S.E.M. (*n* = 3). * *p* < 0.05 vs. CTRL (1), *** *p* < 0.001 vs. CTRL (1), $$ *p* < 0.05 vs. 50 µM (3), $$$ *p* < 0.001 vs. 50 µM (3), ### *p* < 0.001 vs. 50 nM DIM (2). (one-way ANOVA analysis of variance with Tukey’s post hoc analysis).

Given this evidence for the regulation of pAKT levels in PC3 cells, the same experimental protocol described above was used to test the ability of DIM to modulate the activity of CASP-3. Both doses of DIM, 50 nM and 50 µM, induced a significant increase of cleaved CASP-3 level in PC3 cells (Figure 6, lanes 2 and 3) and this increase was antagonized by SR2 in a dose-related manner (Figure 6, lanes 4–7).

## 3. Discussion

The experimental results obtained in our study have clearly demonstrated that the pharmacological effect of DIM was mediated by CB_2_ receptors as shown by [^35^S]GTPγS assay in PC3 and, to a lesser extent, in LNCap cells.

Given the robust effect in PC3 cells, the CB_2_ receptor-mediated effect of DIM was further investigated in this cell line. In the cAMP assay experiments, DIM inhibited FSK-induced cAMP production, inhibited pAKT and enhanced cleavage of CASP-3, all of which were robustly antagonized by the CB_2_ receptor antagonist.

To our knowledge, there are very few previous studies that describe the ability of DIM to interact with CB_2_ cannabinoid receptors, and these used stable CB_2_ receptor-transfected cellular models. Thus, this may be the first study to describe the effects in cells endogenously expressing this receptor and the associated signaling pathways. Specifically, a study conducted in 2009 by Yin et al. [22] showed that DIM was only able to partially activate CB_2_ receptors when tested in a β-arrestin assay. However, our data in cancer cells in both the [^35^S]GTPγS and cAMP assays, clearly indicate DIM actually displays full agonist activity, similar to the well-known full CB_1_/CB_2_ receptor agonist CP 55,940.

The difference in CB_2_ receptor properties between the two studies is likely due to the different cellular models (recombinant vs. naturally expressing cell lines) used, although it may also reflect the different assays used to characterize the pharmacological response of the CB_2_ receptor to DIM. Whatever the reason, our findings suggest that for the pharmacological characterization of potential CB_2_ receptor ligands, systems naturally expressing the receptor represent a more reliable model than recombinant ones when compared to in vivo models [32]. As mentioned above, when tested in both cell lines, DIM behaved as a full CB_2_ receptor agonist and, at least in PC3 cells, the compound was also able to induce apoptosis through a significant reduction of the pAKT level. The latter is important since pAKT is a key kinase in multiple cellular pathways involved in oncogenic and proliferative functions that can regulate androgen receptor levels in prostate cancer cells [34].

The main factors influencing the growth and progression of prostate cancer are the androgen receptor and the PI3K/AKT pathway. Our observations, therefore, confirm previous studies showing that the activation of the CB_2_ receptor was responsible for AKT protein kinase dephosphorylation in addition to the inhibition of the phosphatidylinositol-3-kinase (PI3K)-AKT pathway [35,36], but importantly, it shows that DIM induces the same effects. The glycogen synthase kinase 3 (GSK-3) and other targets are regulated by AKT phosphorylation increased by the phosphatase and tensin homolog (PTEN) tumor suppressor gene inactivation [37]. PTEN has been reported to be lost or mutated in most advanced prostate cancer cases [38] and there are numerous inhibitors in development that target various PI3K cascade nodes, including PI3K, AKT, mTOR [34]. The effect of DIM on PTEN/PI3K-AKT signaling has been reported in other cancer models [39] and our results suggest it invokes the same inhibitory activity in PC3 cells through CB_2_ receptors. On the other hand, PI3K or AKT inhibition might not be enough to significantly reduce tumor size in prostate cancer, and repression of the AR axis is also necessary for maximum effectiveness [37]. In fact, several investigations showed that PI3K and AR signaling interact [40,41]. This may explain the lack of efficacy in LNCaP cell lines, which are thought to be androgen-dependent, requiring androgens for growth. In contrast, PC3 cell lines are thought to be androgen-independent, not requiring or affecting androgen for growth [42]. It is likely that LNCaP cells with a functioning androgen pathway prevent DIM from activating CB2. In LNCaP cells, the DIM-induced inhibition of cell viability was not antagonized by the CB_2_ receptors antagonist, suggesting the possible involvement of other non-cannabinoid receptors in the mechanism. In fact, in LNCaP cells, DIM can inhibit cancer cell proliferation through different and overlapping mechanisms [43,44] and some cannabinoid ligands have been reported to induce apoptosis through a combination of cannabinoid receptor-independent cellular and molecular mechanisms [45]. Our results, obtained by stimulating LNCaP with DIM, show only a weak effect on cell proliferation which may be ascribed to a non-specific mechanism or to a lower expression of CB_2_ receptors in this cell line [45].

In PC3 cells, activation of the CB_2_ receptor can elicit an antiproliferative effect by promoting an increase in ceramide levels [46]. Ceramide generation occurs from sphingomyelin hydrolysis, with cannabinoids having a stimulatory effect in the process [47]. Indeed, increasing ceramide levels can inhibit the AKT-mammalian target of the rapamycin (mTOR) pathway and activate the initiation factors involved in autophagy regulation and endoplasmic reticulum (ER) stress response [27]. Also, increasing levels of ceramide due to ER stress enhancement can trigger the activation of the caspase cascade, leading to apoptosis as well [27]. As cysteine proteases, caspases are formed constitutively in the cells and are normally present as inactive proenzymes [48]. In this context, our results have shown that treating PC3 cells with DIM caused a significantly increased level of the active pro-apoptotic caspase-3 (CASP-3) kinase, confirming the ability of the compound to induce apoptosis in this human prostate cancer cell line [48].

The CB_2_ receptor-mediated AKT dephosphorylation in PC3 cells after DIM administration may suggest a different signaling pathway is involved, one not involving the activation of PI3K, a kinase normally activated by G_βγ_ subunits upon CB_2_ receptor stimulation [49]. Indeed, the PI3K pathway is directly responsible for AKT activation and provides an intrinsic bypass mechanism able to enhance both the survival and proliferation of prostate cancer cells lacking AR signaling, making these cells resistant to androgen therapy [50]. Thus, the inhibitory role of CB_2_ receptors on AKT, possibly through a mechanism independent of PI3K, may represent promising and beneficial effects of DIM that are worth further investigation, considering the lack of demonstrable efficacy in clinical studies where direct PI3K inhibitor drugs were tested. [50].

Our data suggest that PC3 cells exhibit a constitutively active tone by the endocannabinoids on CB_2_ receptors which modulates the AKT pathway. This is demonstrated as the AKT phosphorylation increased significantly above the basal levels when the effect of DIM was antagonized with the highest dose of CB_2_ receptor antagonist [28].

As mentioned above, in human prostate cancer cells, antiproliferative and apoptotic effects can also be mediated by ceramide accumulation and these compounds have been suggested as possible mediators of cannabinoid action, and more specifically, of the endogenous cannabinoid anandamide [27]. Consistent with this, altered levels of intracellular ceramide have been found in both LNCaP and PC3 cells [51].

## 4. Materials and Methods

### 4.1. Culturing and Treatment of Cells

Human prostate cancer cell lines, LNCaP and PC3, were obtained from the European Collection of Animal Cell Cultures. These were cultured in RPMI 1640 medium (Merck Life Science Ltd., Gillingham, UK) containing 10% (*vol:vol*) fetal bovine serum and 1% (*vol:vol*) Penicillin–Streptomycin Solution (10,000 U/mL penicillin and 10 mg/mL streptomycin in 0.9% sodium chloride (Merck Life Science Ltd., Gillingham, UK).

### 4.2. Biochemical Reagents

CP 55,940 (Merck Life Science Ltd., Gillingham, UK), CB_1_ and CB_2_ receptors agonist, CB_1_- and CB_2_- selective inverse agonists, SR 141716 (SR1) and SR 144528 (SR2) (Bio-Techne Ltd., Abingdon, UK), were dissolved in dimethyl sulfoxide (DMSO) and all stored at 10 mM stock solutions (−20 °C).

Appropriate concentrations were freshly prepared from stock solution using a culture medium. DMSO and ethanol diluent controls were also in. For binding experiments, [^35^S] guanosine 5′-O-[gamma-thio] triphosphate (GTPγS) (1250 Ci/mmol) was obtained from PerkinElmer Life Sciences (Stapeley, Nantwich, UK), GTPγS and adenosine deaminase from Roche Diagnostic (Merck Life Science Ltd., Gillingham, UK) and guanosine diphosphate (GDP) and phenylmethylsulfonyl fluoride (PMSF) from Merck Life Science Ltd., Gillingham, UK. All cells and chemicals were handled using the appropriate personal protective equipment. The DIM concentrations used in this study were chosen according to pharmacokinetic studies in animals and previous studies in prostate cancer cell lines [52].

### 4.3. Membrane Preparation

Binding assays with [^35^S]GTPγS were performed with PC3 or LNCaP cell membranes [53,54]. Both PC3 and LNCaP cells were removed from flasks by scraping and then frozen as a pellet at −20 °C until required [55]. Before use in a radioligand-binding assay, cells were defrosted, diluted in Tris buffer (50 mM Tris–HCl, 50 mM Tris-Base) and homogenized. Protein assays were performed using a Bio-Rad Dc kit (Bio-Rad©, Watford, UK).

### 4.4. [^35^S]GTPγS-Binding Assays

The measurement of ligand-stimulated [^35^S]GTPγS binding to cannabinoid CB_1_ or CB_2_ receptors was adapted from methods described previously [55,56]. The assays were carried out with GTPγS-binding buffer (50 mM Tris–HCl, 50 mM Tris-Base, 5 mM MgCl_2_, 1 mM ethylenediaminetetraacetic acid, 100 mM NaCl, 1 mM dithiothreitol and 0.1% bovine serum albumin) in the presence of [^35^S]GTPγS and GDP, in a final volume of 500 μL. Binding was initiated by the addition of [^35^S]GTPγS to the wells. Non-specific binding was measured in the presence of 30 μM GTPγS. The drugs were incubated in the assay for 60 min at 30 °C. The reaction was terminated by a rapid vacuum filtration method using Tris-binding buffer, as described previously [32], and the radioactivity was quantified by liquid scintillation spectrometry. In all the [^35^S]GTPγS-binding assays, we used 0.1 nM [^35^S]GTPγS, 30 μM GDP and a protein concentration of 40 μg per well for PC3 and LNCaP cell membranes. Compounds were stored at −20 °C in DMSO.

### 4.5. Cyclic AMP Assay

The assays were performed using the HitHunter^®^ cAMP assay kit (Eurofins DiscoverX Products, Celle-L’Evescault, France) according to the vendor’s protocol and previous studies [32,57]. Briefly, cells were detached using cell dissociation buffer, counted and seeded at 2 × 10^4^ cells per well in 100 mL of complete medium onto white 96-well plates and incubated at 37 °C and 5% CO_2_ for approximately 24 h before running the experiment. The assays and the drug dilutions were performed in a 1:1 mixture of DMEM and Ham’s F12 medium without phenol red, containing 10 mM of rolipram and forskolin (FSK; 7b-acetoxy-8,13-epoxy-1a,6b,9 atrihydroxylabd-14-en-11-one). Before running the assay, the medium was discarded, and cells were washed with D-MEM/F-12 medium. Then, cells were treated with the assigned drugs (30 μL per well) and incubated for 30 min at 37 °C and 5% CO_2_. Finally, cAMP standards and the appropriate mixture of kit components were added (as described by the manufacturer, Eurofins DiscoverX Products, Celle-L’Evescault, France). Plates were incubated overnight at room temperature in the dark. Chemiluminescent signals were detected on a Synergy HT Multi-Mode Microplate Reader (ThermoFisher Scientific, Loughborough, UK).

### 4.6. Cell Viability Assay

A standard 3-(4,-5-dimethylthiazol-2-yl)-2,-5-diphenyl tetrazolium bromide (MTT) dye reduction assay was used to assess compounds. Briefly, cells were plated in a flat-bottomed 96-well plate at seeding densities of 6 × 10^3^ cells per well for LNCaP cells and 5 × 10^3^ cells per well for PC3 cells. Cells were treated the following day with the appropriate agents for 24 h. Following the incubation period, the MTT solution (5 mg/mL in phosphate-buffered saline) was added and incubated for 4 h. The contents of the wells were removed and replaced with 200 μL DMSO to dissolve the MTT formazan crystals. The plates were immediately read at 570 nm in a multi-well plate reader (DynaTech MR5000; Dynex Technologies Ltd., Chantilly, VA, USA).

### 4.7. Protein Analysis

Cells were homogenized in lysis buffer [20 mM Tris, 0.25 M sucrose, 10 mM ethyleneglycol-bis(aminoethylether)-tetraacetic acid, 2 mM ethylenediaminetetraacetic acid, 1 mM sodium orthovanadate, 25 mM sodium β-glycerophosphate and 50 mM sodium fluoride; pH 7.5]. Prior to use, 0.1% (*vol:vol*), protease Inhibitor Cocktail (Merck Life Science Ltd, Gillingham, UK) was added. A total of 20 μg of protein was electrophoresed through a precast 16% polyacrylamide gel (Thermo Fisher, Oxford, UK) for 2 h and the separated proteins were transferred to nitrocellulose membranes (Bio-Rad, UK), then blocked with 5% (*wt*:*vol*) skimmed milk in Tris-buffered saline with 0.1% (*vol:vol*) Tween 20 (TBST solution) at room temperature and incubated with 1:200 dilution of anti-pAKT (Ser473) antibody (Merck Life Science Limited, Watford, Hertfordshire UK) or 1:200 cleaved-CASP-3 antibody (Insight Biotechnology, UK) at 4 °C overnight. β-Actin (1:20,000) was used as an internal loading control to normalize between lanes during densitometry. The appropriate secondary antibody for pAKT (anti-mouse), cleaved-CASP-3 (anti-mouse) was used at a concentration of 1:5000 (Insight Biotechnology Ltd, Wembley, Middlesex, UK) and incubated at room temperature for 1 h. Proteins were visualized using ECL plus^TM^ chemiluminescent detection kit (Abcam plc, Trumpington, Cambridge, UK), according to the manufacturer’s instructions, and a Fluor S phosphorimager (Bio-Rad©, Watford, UK). The experiments were performed with proteins isolated from three independent extractions.

### 4.8. Data Analysis

Values for half of the maximal effective concentration required to elicit a response (EC_50_), or for the half maximal inhibitory concentration (IC_50_) required to inhibit a response, as well as for maximal effect (E_max_), standard error of the mean (S.E.M.) and 95% confidence limits of EC_50_ and IC_50_, have been calculated by nonlinear regression analysis using the equation for a sigmoid concentration–response curve (GraphPad Prism v5.0, San Diego, CA, USA). Paired Student’s *t*-test or one-way ANOVA analysis of variance (with Tukey’s post hoc analysis) was used where appropriate for cell viability and western blotting data. A value of *p* < 0.05 was taken as being significant.

## 5. Conclusions

Bringing together our results, we can conclude that DIM is a CB_2_ receptor ligand with a potential anti-prostate cancer effect, and differing from classical cannabinoids, is without any psychotropic activity. In particular, DIM can induce a significant anti-apoptotic activity in the androgen-independent human prostate cancer cell line PC3, a cellular model that mimics prostatic small cell neuroendocrine carcinoma (SCNC), an extremely aggressive form of prostate cancer resistant to standard hormonal therapy [58,59]. According to reports, unlike LNCaP cells, PC3 cells do not express AR and their proliferation is androgen-independent, much like SCNC [58]. A more consistent body of evidence is required to confirm our findings and better understand the biological activity of DIM. The promising results obtained in this study represent a step forward in the understanding of the mechanism(s) underlying the DIM anticancer effect and encourage further exploration into the beneficial effect of this compound for the treatment of those forms of prostate cancer insensitive to the androgen treatment.

Based on our results, we have estimated that the concentrations at which DIM is showing an effect on cell viability could be obtained in vivo with an equivalent daily intake of about 60 mg of the compound. However, such daily intakes cannot be achieved by the ingestion of *Brassica* vegetables alone, therefore, administration in form of supplements may be required [60].

## Data Availability

Data is contained within the article.
